# Low prevalence of *Chlamydia trachomatis *infection in asymptomatic young Swiss men

**DOI:** 10.1186/1471-2334-8-45

**Published:** 2008-04-12

**Authors:** David Baud, Katia Jaton, Claire Bertelli, Jean-Pierre Kulling, Gilbert Greub

**Affiliations:** 1 Institute of Microbiology, University Hospital Centre and University of Lausanne, Lausanne, Switzerland; 2 Recruitment Centre of the Swiss Army, Lausanne, Switzerland

## Abstract

**Background:**

Prevalence and risk factors for *Chlamydia trachomatis *infection among young men in Switzerland is still unknown. The objective of the present study was to assess prevalence and risk factors for *C. trachomatis *infection in young Swiss men.

**Methods:**

517 young Swiss men were enrolled in this cross-sectional study during their compulsory military recruitment. Participants completed a questionnaire and gave urine samples which were screened for *C. trachomatis *DNA by PCR. Genotyping of positive samples was done by amplification and sequencing the *ompA *gene.

**Results:**

The prevalence of chlamydial infection among young Swiss male was 1.2% (95% confidence interval [95%CI], 0.4–2.5%). *C. trachomatis *infection was only identified among the 306 men having multiple sexual partner. Although frequent, neither unprotected sex (absence of condom use), nor alcohol and drug abuse were associated with chlamydial infection. Men living in cities were more frequently infected (2.9%, 95%CI 0.8–7.4%) than men living in rural areas (0.5%, 95%CI 0.1–1.9%, p = 0.046). Moreover, naturalised Swiss citizens were more often positive (4.9%, 95%CI 1.3–12.5%) than native-born Swiss men (0.5%, 95%CI 0.1–1.7%, p = 0.003).

**Conclusion:**

In comparison with other countries, the prevalence of chlamydial infection in men is extremely low in Switzerland, despite a significant prevalence of risky sexual behaviour. *C. trachomatis *infection was especially prevalent in men with multiple sexual partners. Further research is required (i) to define which subgroup of the general population should be routinely screened, and (ii) to test whether such a targeted screening strategy will be effective to reduce the prevalence of chlamydial infection among this population.

## Background

*Chlamydia trachomatis *infection is the commonest sexually transmitted bacterial disease in European countries [1,2] and in the United States [3,4]. Although the major impact of disease is on the female genital tract [1,2], men may suffer from urethritis, prostatitis, infertility and Reiter's syndrome [1].

The most frequent risk factors associated with chlamydial infection are related to sexual behaviour, i.e. early age of first intercourse, multiple partners, and inconsistent condom use [1]. Since most infections caused by *C. trachomatis *are asymptomatic, the establishment of screening programs, as already done in some developed countries, is necessary to control the disease [5].

The studies carried out in Europe on *C. trachomatis *infection among young males have shown high rates ranging from 7.8–13.3%[1,6-11]. Similar prevalence were observed in other developed (3.7–5.3% [4,12-14]) and developing countries (3.1–7.9% [15,16]).

However, no data are available from Switzerland. The present cross-sectional study is intended to determine the prevalence of and risk factors for *C. trachomatis *infection among young healthy Swiss males.

## Methods

In this study approved by the ethical committee of the University of Lausanne (n° 177/06), we included all 18–26 years-old Swiss men who presented for their medical entry examination at the Army Recruitment Centre of Lausanne, Switzerland during winter 2006–2007 and who gave written consent for urinary *C. trachomatis *screening. The volunteers completed a questionnaire on sociodemographic, sexual and behavioral risk factors. Our questionnaire was adapted from questionnaires used in previous published studies measuring sexually transmitted infections [6,12,17-19]. Completion of the questionnaire was accurately done, since answers to similar and complementary questions formulated in different ways were 97% and 95% congruent for demographic questions and for questions on their sexual activities, respectively. DNA was extracted from centrifuged first-void urine and analyzed by real-time Taqman PCR, as described [20]. This PCR exhibited an excellent analytical sensitivity of 1 copy per reaction, good intrarun and interrun reliability, and a specificity of 100% when considering Cobas Amplicor as gold standard [20]. Positive samples were then genotyped using *ompA *sequencing [21]. Statistical analyses were performed using STATA (College-Station, USA). Confidence Intervals were calculated using the Daly method [22].

## Results

Among 521 eligible men, four did not complete the questionnaire and were excluded. The sociodemographic characteristics of the 517 remaining volunteers are shown in Table 1. The mean age was 20.6 years (standard deviation ± 1.4 years). A total of 87% of the participants reported being sexually-experienced and 59.9% of the participants reported having more than two sexual partners in their life span. The consistent use of condoms for all sexual relations was reported by 32.3% of the 517 volunteers. Among those with more than 4 partners (n = 178), 21.3% regularly used condoms. While 67.9% of the study participants drank alcohol at least every week-end, 3.5% drank alcohol every day. Almost half (47.6%) of the participants were cigarette smokers, and 28.1% of them smoke more than 20 cigarettes per day. Occasional or daily cannabis consumption was reported by 27.1% and 7.8% of the participants, respectively. Ecstasy, heroin or cocaine have been taken by 6.4% of the studied population.

**Table 1:** *Chlamydia trachomatis *prevalence according to various demographic and behavioural variables

**Characteristics**	*C. trachomatis *negative	(%)	*C. trachomatis *positive	(%)	p-value*	Odds Ratio	95% CI	Prevalence of *C. trachomatis*	Daly 95% CI
**Total**	511	98.8	6	1.2				1.2	0,4 – 2,5
**Age (year ± SD)**	20.6 + 1.4		20.7 + 2.7						
≤ 20	276	54	5	83.3	0.23	ref		1.8	0.6 – 4.2
> 20	235	46	1	16.7		0.23	0,03 – 2,02	0.4	0 – 2.4
**Nationality at birth**									
Switzerland	433	84.7	2	33.3	0.003	ref		0.5	0.1 – 1.7
Europe	41	8	1	16.7		5.3	0,47 – 59,5	2.4	0.1 – 13.3
Other	37	7.2	3	50		17.6	2,84 – 108	7.5	1.5 – 21.9
**Place of residence**									
> 10'000 inhabitants	134	26.2	4	66.7	0.046	ref		2.9	0.8 – 7.4
< 10'000 inhabitants	377	73.8	2	33.3		0.17	0.03 – 0.98	0.5	0.1 – 1.9
**Main occupation**									
Work	265	51.9	4	66.7	0.74	ref		1.5	0.4 – 3.8
Studies	231	45.2	2	33.3		0.53	0,1 – 2,69	0.9	0.1 – 3.1
Declined to respond	15	2.9	0	0		-	-	0	0 – 20
**Monthly income**									
< 1000 Frs.	301	58.9	1	16.7	0.064	ref		0.3	0 – 1.8
1000 – 2000 Frs.	69	13.5	1	16.7		4.4	0,3 – 70,6	1.4	0 – 8
> 2000 Frs.	112	21.9	4	66.7		10.8	1,2 – 97,2	3.5	0.9 – 8.8
Declined to respond	29	5.7	0	0		-	-	0	0 – 10,3

**Number of lifetime sexual partner**									
0	67	13.1	0	0	0.26	-	-	0	0 – 4.5
1	125	24.5	0	0		-	-	0	0 – 2,4
≥ 2	306	59.9	6	100		-	-	1.9	0.7 – 4.2
Declined to respond	13	2.5	0	0		-	-	0	0 – 23
**Sexual orientation**									
heterosexual	386	75.5	4	66.7	0.66	ref		1	0.3 – 2.6
homo/bisexual	9	1.8	0	0		-	-	0	0 – 33,3
Declined to respond	116	22.7	2	33.3		1.7	0,3 – 9,2	1.7	0.2 – 6.1
**Condom use**									
Always	165	32.3	2	33.3	1	ref		1.2	0.1 – 4.3
Sometimes	238	46.6	3	50		1.04	0,17 – 6,29	1.2	0.3 – 3.6
Never	74	14.5	1	16.7		1.11	0,10 – 12,5	1.3	0 – 7.4
Declined to respond	34	6.7	0	0		-	-	0	0 – 8.8

* Fisher's exact chi^2^

The prevalence of chlamydial infection was 1.2% (95%CI 0.4–2.5%) among all 517 volunteers and of 1.3% (95%CI 0.3–2.4%) among the 450 sexually-experienced men. No participants without or with only a single lifetime sexual partner were diagnosed with chlamydial infection. Conversely, 1.9% (95%CI 0.7–4.2%) of those with multiple sexual partners were *C. trachomatis *positive. Two men who tested positive for *Chlamydia *reported having consistently used condoms during all sexual relations. Among the 9 men who reported having had a male sexual partner, none had a positive *Chlamydia *test. When considering only Swiss born men, the prevalence was 0.5% (95%CI 0.2–2.1%). Conversely, among foreign born naturalized men who originated from Europe, the prevalence was 2.4% (95CI 0.1–13.3%). For those originated from foreign countries outside Europe, a prevalence of 7.5% (95%CI 1.5–21.9%) was observed. Young men living in cities more than 10'000 inhabitants and/or earning more than 2000 SFrs (1600 US$) were at increased risk of *C. trachomatis *infection (Table 1). Alcohol, cigarette, cannabis and illegal drug consumption were frequently recorded, but were not associated with chlamydial infection. Chlamydial serotypes were E (n = 3), J, Ia and D (n = 1 each).

## Discussion

Our cross-sectional study provides, for the first time, prevalence data of *C. trachomatis *infection in young Swiss men. In Switzerland, previous studies mainly focused on women and evaluated highly selected study populations [23]. A 2.8% prevalence was recorded among asymptomatic sexually active Swiss women in 1998 [23]. However, studied women were mainly from urban area, where the prevalence is higher. Moreover, age of first sexual intercourse is lower for women or occur with older partner [24], likely also contributing to increased prevalence in women. *C. trachomatis *is a reportable disease in Switzerland. Since 1999, there has been a significant increase (64%) in the number of official reports of *C. trachomatis *infection in Switzerland [25,26]. However, among the 517 asymptomatic young Swiss men we investigated, the prevalence was only 1.2%. This *Chlamydia *prevalence is much lower than that reported for young asymptomatic males in Norway (7.8%)[6], Scotland (9.8%) [8] and the entire UK (13.3%) [1]. In USA, prevalence is also higher than that found in Switzerland, ranging from 3.7% [4] to 5.3% [13].

Multiple partners and inconsistent condom use are recognised as important predictors of *C. trachomatis *infection [1,6,16]. However, screening on the basis of condom use would have missed a substantial number of infections in the population we studied, since two of six infected men reported having always used condoms. The effect or no effect of condom use as a preventive mesure should ideally be confirmed in a larger cohort. In our study, variables associated with *C. trachomatis *infection were "multiple sexual partners", "living in a large city" and "foreign born, naturalized". The higher prevalence observed in the latter subgroup, similar to that of other European and non-European countries, demonstrate that the overall low prevalence of 1.2% we observed was not due to a low sensitivity of the PCR, but rather reflects a true local difference in prevalence. Serotype E was more prevalent in Switzerland, as it is the case in other European countries [21,27].

## Conclusion

This is the first study of the prevalence of *C. trachomatis *infection conducted in young Swiss males. Since military service is compulsory for all Swiss men, the study population we screened corresponds to an unselected and unbiased representative sample of young healthy Swiss men. Despite a prevalence of sexual risk behaviour which is similar to that recorded in other countries [24], *C. trachomatis *prevalence in Swiss males is extremely low. In the future, in order to identify populations with a high prevalence that may benefit from mass screening, it will be important to more precisely identify risks factors associated with chlamydial infection.

## Competing interests

The author(s) declare that they have no competing interests.

## Authors' contributions

DB and GG initiated and designed the study, interpreted the results. DB, CB and GG performed the statistical analyses. DB and JPK were responsible for patient recruitment, clinical assessment, data management and blood sampling. KJ and GG did the laboratory analyses. The paper was written by DB and GG, and reviewed by all other contributors.

## Pre-publication history

The pre-publication history for this paper can be accessed here:


